# Influence of Alpine Forest Types on Soil Microbial Diversity and Soil Quality

**DOI:** 10.3390/plants15020315

**Published:** 2026-01-21

**Authors:** Shuang Ji, Xunxun Qiu, Huichun Xie, Zhiqiang Dong, Hongye Li

**Affiliations:** 1 College of Geographical Sciences, Qinghai Normal University, Xining 810008, China; 202247341022@stu.qhnu.edu.cn (S.J.);; 2 Qilian Mountain Southern Slope Forest Ecosystem Research Station, Huzhu 810500, China; 3 Key Laboratory of Medicinal Animal and Plant Resources of Qinghai-Tibetan Plateau, Xining 810008, China; 4 Team of Germplasm Resources Formation Mechanism and Utilization on the Qinghai-Tibetan Plateau, Xining 810008, China; 5 School of Life Sciences, Qinghai Normal University, Xining 810008, China; 6 Innovation and Intelligence Introduction Base for Plateau Resources Ecology and Sustainable Development, Xining 810008, China

**Keywords:** forest types, soil microbial diversity, soil quality, southern slope of the Qilian Mountains

## Abstract

Alpine forests are key regulators of soil biogeochemical cycles, yet the extent to which forest type constrains soil microbial diversity and soil quality in high-elevation regions remains insufficiently resolved. Here, we assessed how contrasting alpine forest types influence the taxonomic composition and diversity of soil microbial communities, identified the dominant environmental drivers, and evaluated soil quality along the southern slope of the Qilian Mountains. Six forest types were examined, including four monospecific stands (*Picea crassifolia*, QQ; *Betula* spp., HS; *Juniperus przewalskii*, YB; and *Pinus tabuliformis*, YS) and two mixed formations (mixed conifer–broadleaf, ZKHJ; and mixed broadleaved, KKHJ). Bacterial and fungal communities were characterized using Illumina high-throughput sequencing, while structural equation modeling (SEM) was used to identify primary drivers of diversity and principal component analysis (PCA) was applied to construct the minimum data set (MDS) for soil quality evaluation. Mixed forests consistently exhibited higher bacterial and fungal alpha diversity than pure stands. Environmental gradients were the strongest positive drivers of microbial diversity, whereas soil chemical properties and vegetation-related biotic factors exerted partially negative effects. Soil quality index (SQI) values ranked as follows: KKHJ (0.55) > ZKHJ (0.49) > YB (0.48) > HS (0.46) > YS (0.44) > QQ (0.43). The mixed broadleaved forest reached Grade IV (upper-intermediate level) soil quality, whereas the other forest types were classified as Grade III (intermediate). Mixed forests showed stronger capacities for organic matter accumulation and nutrient retention. These findings indicate that promoting mixed forest stands is critical for improving soil structure, nutrient retention, and microbial diversity in this alpine region. Accordingly, forest management should prioritize the development of mixed forests to enhance overall soil quality.

## 1. Introduction

Forests represent a fundamental element of terrestrial ecosystems and exert a pivotal influence on global carbon and nitrogen cycling [1,2]. The quality of their soils, which serve as major reservoirs of terrestrial organic matter, directly influences global nutrient cycling, carbon sequestration potential, ecosystem stability, and biodiversity [3]. Within forest ecosystems, the interplay between soil physicochemical attributes and microbial assemblages is fundamental to sustaining soil functionality and overall ecosystem stability. Soil quality (SQ) has, in recent years, been widely adopted as a comprehensive measure of soil health, integrating physical, chemical, and biological attributes to provide a robust indication of soil functioning under distinct land-use and management regimes [4]. The soil quality index (SQI) has been extensively applied across diverse ecosystems, including forests and farmlands, providing a quantitative evaluation framework for soil health and sustainable use [5].

Different forest types shape distinct soil environments by altering litter input, root exudates, and microclimate conditions [6], thereby strongly influencing soil nutrient supply capacity, microbial activity, and the stability of soil organic carbon (SOC) and total nitrogen (TN) pools [7]. Accordingly, comparing the physicochemical properties and soil quality (SQ) characteristics of soils across forest types is essential for elucidating the functional role of forest vegetation in regulating soil carbon and nitrogen cycling and associated nutrient maintenance mechanisms. Soil quality is commonly evaluated using integrated indicator systems that combine physical, chemical, and biological attributes, such as bulk density, soil moisture, nutrient availability, organic matter content, enzyme activity, and microbial diversity. To reduce indicator redundancy and improve interpretability, multivariate approaches—particularly minimum data set (MDS) selection based on principal component analysis (PCA)—are widely applied to construct soil quality indices (SQI). Based on SQI values, soil quality can be classified into six grades (I–VI): Grade I (SQI ≤ 0.30), Grade II (0.30 < SQI ≤ 0.40), Grade III (0.40 < SQI ≤ 0.50), Grade IV (0.50 < SQI ≤ 0.60), Grade V (0.60 < SQI ≤ 0.70), and Grade VI (SQI > 0.70) [8]. Soil microorganisms, particularly fungi and bacteria, serve as the primary drivers of soil carbon and nitrogen transformations, participating in key processes such as organic substrate decomposition, nutrient mineralization, biological nitrogen acquisition, and nitrogen loss via denitrification [9]. Environmental factors, including soil pH, moisture content, and nutrient availability, substantially influence microbial community composition and diversity [10]. Moreover, functional microbial guilds mediating carbon and nitrogen cycling (e.g., decomposers, nitrogen-fixing bacteria, and denitrifiers) provide critical insights into soil fertility maintenance and ecosystem stability [11]. Despite this importance, previous studies have predominantly examined soil quality and microbial diversity within single forest types or across limited ecological gradients, whereas comprehensive comparisons across multiple forest types within the same region remain scarce, particularly those integrating soil physicochemical properties, carbon and nitrogen pools, and microbial community structure, thereby constraining a mechanistic understanding of the coupling between soil quality and microbial diversity in alpine forest ecosystems.

This study aimed to investigate how different forest types regulate soil microbial community composition, diversity, and soil quality across six forest ecosystems in the Huzhu Beishan region of the northeastern Qinghai–Tibet Plateau. Specifically, we asked whether bacterial and fungal community structures and diversity differ among forest types, and how soil physicochemical properties and microbial attributes jointly influence soil quality formation. We hypothesized that mixed forest stands would support higher microbial diversity and improved soil quality compared with pure stands due to more favorable soil structure and nutrient conditions. By integrating soil physicochemical indicators with microbial assemblage data, this study provides mechanistic insights into soil quality formation in forest ecosystems and offers a scientific basis for regional forest management, soil health conservation, and carbon–nitrogen balance regulation.

## 2. Materials and Methods

### 2.1. Study Area

The study area is located on the southern flank of the Qilian Mountains in Qinghai Province, positioned in the northeastern sector of the Qinghai–Tibet Plateau. Geographically, it spans 98°08′13″–102°38′16″ E and 37°03′17″–39°05′56″ N, encompassing roughly 24,000 km^2^. Elevation varies markedly from 2286 to 5210 m, with a mean altitude of approximately 3800 m. The region receives 2200–2900 h of annual sunshine and is characterized by a low mean annual temperature of about −5.9 °C. Warmer temperatures and increased precipitation coincide with the plant-growing season, during which the majority of rainfall—typically 300–400 mm annually—occurs between June and August. Climatically, the environment exhibits the hallmarks of alpine systems, featuring prolonged, severe winters and brief, cool summers. Topographically, the landscape is dominated by mountainous terrain with pronounced elevation gradients. Interactions between its heterogeneous topography and substantial climatic variability give rise to clear vertical vegetation zonation, comprising grasslands, shrublands, and forest belts [12]. The forest vegetation in this region is dominated by cold-temperate coniferous species, including representatives of *Picea*, *Juniperus*, and *Pinus*. In addition to these conifers, several deciduous broadleaf genera occur, such as *Betula* and *Populus*, accompanied by other birch taxa. The local pedological spectrum spans a range of soil types, from mountain forest and cinnamon soils to calcareous kastanozems, chernozems, alpine meadow soils, and even alpine desert substrates [13]. Although multiple soil types occur across the region, all sampling sites in this study were located on gray-brown soils, thereby minimizing the influence of soil type variability on the observed patterns.

### 2.2. Soil Sample Collection and Processing

**Figure 1:**
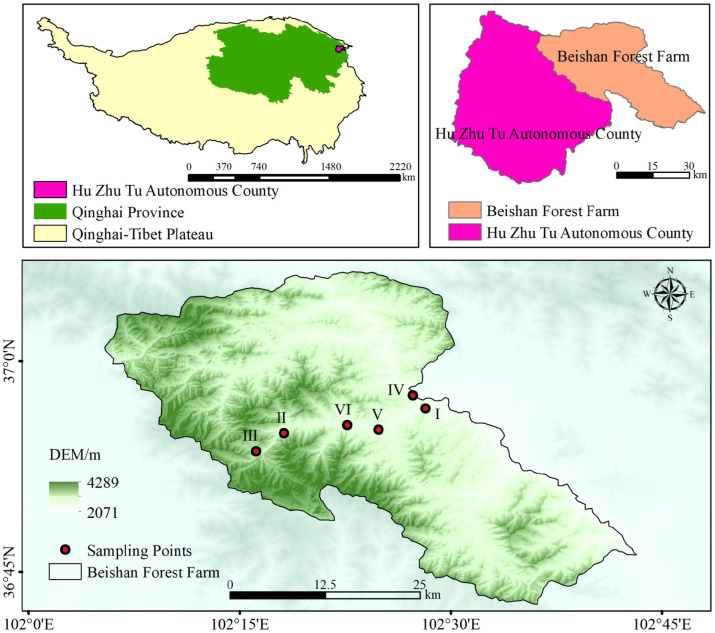
Overview of the Study Area.

Field sampling was conducted during July–August 2024 in forested areas of Huzhu Beishan, located on the southern margin of the Qilian Mountains. The survey encompassed six forest formations: four single-species stands-*Picea crassifolia*, *Betula* spp., *Juniperus przewalskii*, and *Pinus tabuliformis*-and two mixed assemblages dominated by conifer-broadleaf or broadleaf taxa (Figure 1; Table 1). For each forest type, three square plots (20 m × 20 m) were established under similar site conditions, resulting in a total of 18 plots. All woody individuals with a diameter at breast height (DBH) ≥ 3 cm were inventoried, and DBH, tree height, and crown dimensions were recorded. To reduce spatial dependence among plots, a minimum distance of 100 m was maintained. Each plot was subdivided into three 10 m^2^ subplots, within which soil samples were collected along an S-shaped transect. Five soil cores were extracted from the 0–20 cm layer using a soil auger and subsequently pooled to form one composite sample per subplot [14]. In total, 54 composite soil samples were obtained. Each composite soil sample collected for physicochemical analyses was divided into two subsamples: one fresh portion for soil enzyme activity assays and another air-dried portion for physicochemical measurements after removing coarse fragments and undecomposed litter. Soil samples for microbial analyses were collected independently from separate, dedicated soil cores rather than from the composites used for physicochemical analyses. Microbial sampling followed the same spatial sampling design described above: within each subplot, five soil cores were collected from the 0–20 cm layer along an S-shaped transect using a soil auger and then pooled to form one composite microbial sample per subplot. Throughout microbial sampling, all tools directly contacting soil (including augers, sieves, and centrifuge tubes) were strictly sterilized according to standard microbiological sampling protocols to avoid contamination. Immediately after collection, microbial composite samples were transferred into sterile centrifuge tubes, flash-frozen in liquid nitrogen, and stored at −80 °C until DNA extraction. These samples were used for subsequent analyses of soil microbial diversity and community composition. In total, 54 microbial composite samples were collected.

### 2.3. Determination of Soil Physicochemical Properties

The physicochemical properties of the soil were determined following the analytical guidelines provided in the Soil Agrochemical Analysis Manual [15]. Particle size distribution was measured using a laser diffraction system (Mastersizer 2000, Malvern Instruments, Malvern, UK) and classified according to the USDA soil texture classification system. Moisture content was derived gravimetrically, whereas bulk density was calculated from undisturbed cores extracted with a ring-knife sampler. Electrochemical parameters were measured using dedicated instruments: electrical conductivity with a LEICI conductivity meter (LEICI, Shanghai Leici Scientific Instruments Co., Ltd., Shanghai, China) and soil pH with a Sartorius pH meter (Sartorius, Göttingen, Germany). Carbon- and nitrogen-related indices (TC, SOC, TN) were quantified using an elemental analyzer (FlashSmart, Thermo Fisher Scientific, Waltham, MA, USA). Phosphorus fractions (TP and AP) were evaluated via the antimony–molybdenum blue spectrometric method, and potassium fractions (TK and AK) were determined after NaOH fusion followed by flame photometry. Alkali-hydrolysable nitrogen (AN) was assessed using an alkali-diffusion approach. Microbial biomass indicators (MBC, MBN, MBP) were quantified through the chloroform fumigation–extraction technique. The activities of the principal soil enzymes—SUC, URE, ALP, and CAT—were determined following the analytical methods provided by Cui et al. [16].

### 2.4. Analysis of Soil Microbial Diversity

Genetic material was isolated from soil samples with the E.Z.N.A.^®^ Soil DNA Kit (Omega Bio-tek, Norcross, GA, USA) following the standard extraction workflow. DNA integrity was verified by running aliquots on 1% agarose gels, and NanoDrop 2000 measurements (Thermo Fisher Scientific, Waltham, MA, USA) were used to assess nucleic acid purity. Bacterial profiling targeted the V3–V4 region of the 16S rRNA gene, amplified using primer sets 338F (5′-ACTCCTACGGGAGGCAGCAG-3′) and 806R (5′-GGACTACHVGGGTWTCTAAT-3′), each tagged with sample-specific barcodes [6]. Fungal communities were assessed by amplifying the ITS2 region with gITS7 (5′-GTGARTCATCGARTCTTTG-3′) and ITS4 (5′-TCCTCCGCTTATTGATATGC-3′). Amplified fragments were visualized on 2% agarose gels, after which they were purified using a commercial PCR Clean-Up Kit (YuHua Biotech, Shanghai, China). Amplicon yields were quantified with a Qubit 4.0 fluorometer (Thermo Fisher Scientific, Waltham, MA, USA) using the Qubit dsDNA HS Assay Kit. Sequencing libraries were subsequently generated and processed on an Illumina PE300/PE250 platform operated by Majorbio Bio-Pharm Technology Co., Ltd. (Shanghai, China).

### 2.5. Bioinformatics Processing of Bacterial and Fungal Sequencing Data

Raw paired-end reads were quality-filtered using fastp (v0.19.6), in which low-quality bases (Phred < 20) were trimmed using a 50 bp sliding window, and reads shorter than 50 bp or containing ambiguous bases were discarded. High-quality reads were merged using FLASH (v1.2.11) with a minimum overlap of 10 bp and a maximum mismatch ratio of 0.2. Quality-controlled sequences were clustered into operational taxonomic units (OTUs) at 97% similarity using UPARSE (v7.1), during which chimeric sequences were removed and non-target chloroplast and mitochondrial sequences were excluded. To minimize the effects of uneven sequencing depth, all samples were rarefied to 20,000 reads, resulting in an average Good’s coverage of 99.09%. Representative OTU sequences were taxonomically assigned using the RDP Classifier (v2.11) against the SILVA 16S rRNA database (v138) for bacteria and the UNITE database for fungi, with a confidence threshold of 70%.

### 2.6. SQ Evaluation

This study employed 24 indicators to assess SQ, facilitating a comprehensive evaluation of the dimensionality reduction of the full dataset and integrated SQI. Principal component analysis (PCA) was subsequently applied to classify the indicators into component clusters. Within each component cluster, variables whose Norm values fell within 10% of the maximum Norm value were retained, and their composite loads was then calculated to derive the minimum data set (MDS). Among all PCs, a higher composite loading denotes a stronger contribution to explaining integrated system variability [17,18]. The composite loading was calculated using the following expression:

(1) $$N_{\mathrm{ik}}=\sqrt{\sum_{1}^{k}\left(U_{\mathrm{ik}}^{2}\lambda_{k}\right)}$$

where N_ik_ denotes the aggregated loading of indicator i summed across all k principal components; U_ik_ represents the loading of indicator i on an individual principal component; λ_k_ corresponds to the eigenvalue associated with the kth principal component.

Membership functions between these indicators and SQ were established to calculate the membership values for indicators included in the MDS [8]. The calculation formula is presented below:

(2) $$u\left(x\right)=\left\{0.9\times \begin{array}{c} 1,\;x\geq x_{2}\\ \frac{x-x_{1}}{x_{2}-x_{1}},\;x_{2}<x\;\leq x_{1}\;\;\\ 0,x\leq x_{1} \end{array}\right.$$

where x denotes the observed value of a given SQ indicator; x_1_ and x_2_ represent its minimum and maximum values, respectively; and u(x) refers to the standardized form of the indicator.

The SQI was calculated by summing the weighted linear scores of the indicators based on their respective weighting factors, as shown in the following equation [18]:

(3) $$\mathrm{SQI}=\sum_{i=1}^{n}W_{i}N_{i}$$

where N_i_ and W_i_ denote the linear score and weighting coefficient assigned to the ith indicator, respectively, and *n* represents the total number of soil indicators included in the dataset.

### 2.7. Statistical Analysis

Alpha-diversity indices were computed with Mothur (http://www.mothur.org/wiki/Calculators (accessed on 15 January 2026). Differences in alpha diversity among forest categories were examined using the Wilcoxon rank-sum test. Community compositional variation was quantified through the Bray–Curtis dissimilarity metric. Variation in soil properties and microbial traits was analyzed using one-way ANOVA, and when significant, Tukey’s HSD post hoc test was applied. All measured variables were incorporated into the principal component analysis (PCA). Statistical analyses were carried out using SPSS Statistics 26 (IBM Corp., Armonk, NY, USA), R version 4.3.1 (R Core Team, Vienna, Austria), and Origin 2025 (OriginLab Corp., Northampton, MA, USA).

## 3. Results

### 3.1. Differences in Soil Physicochemical Properties and Carbon-Nitrogen Content

The physicochemical attributes of the soil within the 0–20 cm soil layer varied markedly across the six forest types. As shown in Table 2, the mixed stands (ZKHJ and KKHJ) displayed higher TC, TN, SOC, AN, AP, AK, MBC, MBN, and MBP contents than other forest types. In contrast, YS demonstrated lower soil TC, TN, TP, SOC, AN, and AK concentrations, suggesting potential limitations in bioavailable carbon and nitrogen nutrients. YB showed the highest TP content, significantly exceeding those observed in KKHJ, QQ, HS, and YS (*p* < 0.05). Within the entire forest assemblage, YB had the greatest TK concentration, with TK levels at the YB, YS, and QQ coniferous sites substantially exceeding corresponding values in the three broadleaf sites. KKHJ showed the greatest SUC and ALP activities across the soil layer, indicating that mixed broadleaved stands possessed markedly greater carbon and phosphorus transformation potential than pure forests. YS showed the highest soil URE and CAT activities, likely reflecting a stress-induced enzymatic response under low nutrient conditions. Specifically, its URE activity surpassed those measured in QQ, HS, YB, and ZKHJ to a significant extent, and its CAT activity significantly exceeded the levels recorded in HS, YB, ZKHJ, and KKHJ. However, the SUC activity of YS was markedly lower relative to that of the remaining five stand types (*p* < 0.05), indicating weaker carbon transformation potential. HS and YB displayed comparatively low URE and CAT activities, suggesting weaker nitrogen activation and antioxidant enzyme systems.

KKHJ and ZKHJ exhibited higher SWC than each of the four pure forest types. SWC reached its maximum in KKHJ and its minimum in YS. In both cases, the observed values were significantly different from the values recorded for the remaining forest categories (*p* < 0.05), being higher in KKHJ and lower in YS. YS had the greatest BD, markedly exceeding the levels observed in HS, ZKHJ, and KKHJ (*p* < 0.05). YB exhibited the lowest EC levels, which were distinctly reduced compared with the values of the five other stand types (*p* < 0.05), reflecting lower mineral ion activity. Soil pH values across different forests in the study area spanned 6.05–7.89, averaging 7.08, thereby indicating slightly alkaline conditions. YS recorded the greatest pH value, significantly surpassing that of the remaining five forest types (*p* < 0.05). The soil clay content of QQ, YB, and ZKHJ markedly exceeded the level observed in KKHJ (*p* < 0.05). The silt content of YS substantially surpassed the proportions measured in HS, ZKHJ, and KKHJ, whereas the sand fraction in KKHJ was appreciably greater than that in QQ, YB, and YS (*p* < 0.05).

### 3.2. Taxonomic Composition and Diversity of Soil Microbial Communities

**Figure 2:**
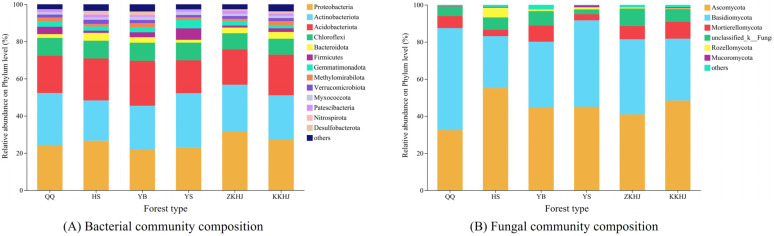
Relative abundance of dominant microbial phyla in six forest types on the southern slope of the Qilian Mountains: (**A**) bacterial community composition and (**B**) fungal community composition. Only phyla with relative abundance >1% are shown; others are grouped as “others.”.

Across the six forest stands on the southern slope of the Qilian Mountains, the taxonomic profiles of both bacterial (Figure 2A) and fungal (Figure 2B) communities differed markedly. Bacterial communities were dominated by members of *Proteobacteria*, *Actinobacteriota*, and *Acidobacteriota*, though the proportional contribution of these phyla varied among forest types. Specifically, *Proteobacteria* dominated in HS, YS, and ZKHJ; *Actinobacteriota* was predominant in QQ and ZKHJ; *Acidobacteriota* was more abundant in YB and KKHJ. Certain groups, including *Chloroflexi* and *Bacteroidota*, though less abundant, still exhibited differences in abundance across forest types. Fungal assemblages were chiefly structured by *Ascomycota* and *Basidiomycota*, which collectively held absolute dominance; however, their proportions showed significant differentiation. *Ascomycota* were more abundant in HS, YB, and KKHJ, while *Basidiomycota* dominated in QQ and ZKHJ. *Mortierellomycota* occurred at low abundance in several forest types, while other fungal groups were extremely rare.

**Figure 3:**
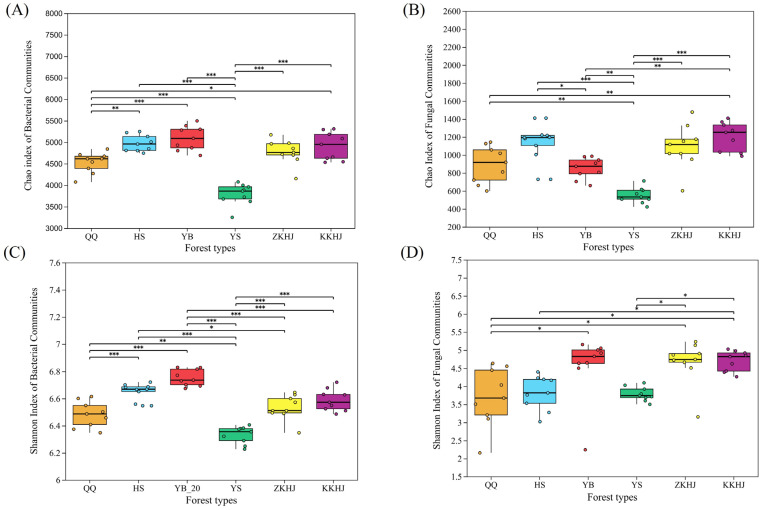
alpha diversity indices of soil microbial communities in six forest types on the southern slope of the Qilian Mountains: (**A**) Chao index of bacterial communities, (**B**) Chao index of fungal communities, (**C**) Shannon index of bacterial communities, and (**D**) Shannon index of fungal communities. Significant differences among forest types are indicated by *p* < 0.05 (*), *p* < 0.01 (**), and *p* < 0.001 (***).

Across the southern flank of the Qilian Mountains, the alpha diversity metrics characterizing soil microbial assemblages demonstrated significant differences across the six forest types (Figure 3). Regarding bacterial communities (Figure 3A,C), HS and YB exhibited higher Chao richness and Shannon diversity metrics, reflecting greater species richness and enhanced taxonomic richness as well as community heterogeneity. In contrast, YS exhibited significantly lower Chao and Shannon values, implying a comparatively simple community structure. Mixed forest types (ZKHJ and KKHJ) exhibited moderate levels for both indices, with KKHJ showing slightly higher bacterial diversity than ZKHJ. For fungal communities (Figure 3B,D), a similar but inconsistent pattern was observed. KKHJ and ZKHJ displayed the highest Chao and Shannon indices, indicating that mixed forest environments significantly promoted fungal abundance and diversity. Conversely, YS exhibited the lowest fungal diversity, while QQ and HS showed moderate levels. Although YB demonstrated relatively high fungal Shannon indices, its Chao indices were lower compared to HS and mixed forest types. Overall, mixed forests supported higher fungal alpha diversity, whereas HS and YB pure forests maintained higher bacterial alpha diversity.

**Figure 4:**
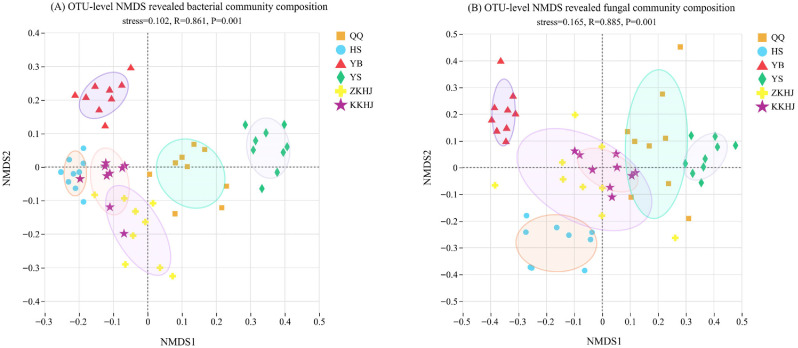
Non-metric multidimensional scaling (NMDS) analysis based on OTU level showing differences in (**A**) bacterial and (**B**) fungal community composition across six forest types on the southern slope of the Qilian Mountains. Ellipses represent 95% confidence intervals for each forest type.

Non-metric multidimensional scaling (NMDS) ordinations based on OTU data revealed distinct partitioning of bacterial and fungal communities among the six forest types on the southern slope of the Qilian Mountains (Figure 4A,B). The resulting NMDS ordination of bacterial assemblages (stress = 0.102, R = 0.861, *p* = 0.001) showed clear two-dimensional distributions of various forest types with high inter-community separation. Specifically, YS and YB exhibited relatively independent distributions, whereas HS, ZKHJ, and KKHJ communities showed some overlap but remained distinctly separable. The NMDS ordination of fungal communities (stress = 0.165, R = 0.885, *p* = 0.001) similarly revealed significant differentiation in community composition across forest types. The fungal communities of YB and YS showed distinct clustering in the coordinate space, which differed from other forest types; ZKHJ and KKHJ partially overlapped while remaining distinguishable from HS and QQ. Overall, fungal assemblages displayed a clearer degree of compositional differentiation between forest types than bacterial communities, suggesting that fungal community structure is more responsive to forest type.

### 3.3. Analysis of Driving Factors for Soil Bacterial and Fungal Diversity

**Figure 5:**
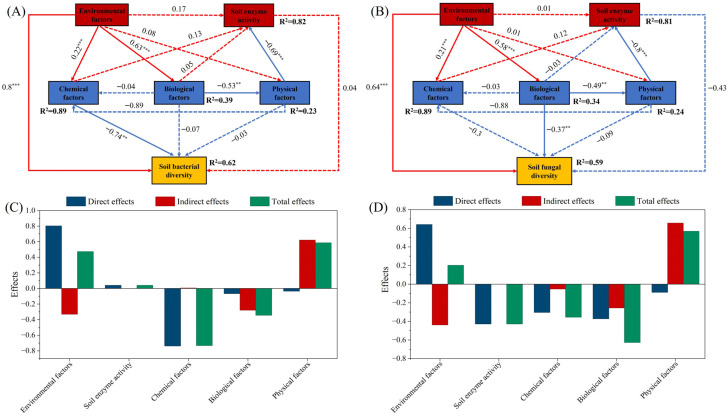
The structural equation model illustrates the driving factors of soil bacterial (**A**) and fungal diversity (**B**). The numbers on the arrows indicate path coefficients, with solid and dashed arrows representing significant and non-significant relationships, respectively. R^2^ represents the proportion of explained variance. Significance levels: ** *p* < 0.01, *** *p* < 0.001. The bar charts show the standardized effects (total effects, direct effects, and indirect effects) of each influencing factor on soil bacteria (**C**) and soil fungi (**D**) derived from the structural equation model.

To assess key determinants in shaping soil bacterial and fungal diversity, we applied structural equation modeling (SEM) to examine how environmental variables (elevation, slope, aspect), soil chemical traits (TC, TN, TP, TK, SOC, AN, AP, AK, pH, MBC, MBN, MBP), soil physical properties (SWC, BD, EC, clay, silt, sand), soil enzyme activities (SUC, URE, ALP, CAT), and vegetation-related biotic factors (forest type, tree height, DBH, crown spread) influence soil bacterial and fungal diversity. The models explained 62% and 59% of the variation in bacterial and fungal diversity, respectively (Figure 5A,B; Table S1), demonstrating a good model fit (GOF = 0.68 for bacteria, 0.67 for fungi; all GOF > 0.6).

The structural equation model for soil bacterial diversity (Figure 5A,C; Table S1) showed that environmental factors exerted a strong and significant direct positive effect on bacterial diversity (*λ* = 0.8, *p* < 0.001). In contrast, soil chemical properties had a significant direct negative effect (*λ* = −0.74, *p* < 0.01). The direct effects of soil enzyme activity on bacterial diversity were positive but not significant (*λ* = 0.04, *p* > 0.05). In addition, biotic factors and soil physical properties influenced bacterial diversity indirectly, although these effects were weak and not statistically significant (biotic factors: *λ* = −0.07, *p* > 0.05; soil physical properties: *λ* = −0.03, *p* > 0.05). The structural equation model for soil fungal diversity (Figure 5B,D) indicated that environmental factors also had a significant direct positive effect on fungal diversity (*λ* = 0.64, *p* < 0.001). Biotic factors showed a significant direct negative influence on fungal diversity (*λ* = −0.37, *p* < 0.01). By contrast, the direct effects of soil chemical properties and soil enzyme activity on fungal diversity were negative but not statistically significant (soil chemical properties: *λ* = −0.3, *p* > 0.05; soil enzyme activity: *λ* = −0.43, *p* > 0.05). Soil physical properties affected fungal diversity indirectly, and this indirect effect was also negative and non-significant (*λ* = −0.09, *p* > 0.05).

### 3.4. Comprehensive SQ Evaluation

#### 3.4.1. Establishment of MDS Based on PCA

PCA and correlation analysis were conducted on the 24 indicators (comprising 22 soil physicochemical attributes and bacterial/fungal diversity metrics) to identify key indicators for SQ assessment. For the *Picea crassifolia* pure forest of the Huzhu Beishan Forest Farm situated along the southern flank of the Qilian Mountains, the two principal components (PC1 and PC2) with eigenvalues >1 accounted for 95.77% of the total variance (Table S2). A larger factor loading value of an indicator suggests greater weight in the principal components and a stronger influence on soil. The soil indicators in PC1 included TC, TN, TP, SOC, AK, MBC, MBN, MBP, URE, ALP, SWC, BD, clay, silt, sand, bacterial diversity, and fungal diversity. The soil indicators in PC2 included TK, AN, AP, SUC, CAT, EC, and pH. All soil indicators were normalized to dimensionless values to enable comparison across variables with different units. The resulting Norm values represent standardized relative magnitudes of each indicator, and those within 10% of the maximum Norm value were retained for further analysis [8]. The ranking of Norm values in PC1 was as follows: MBN > TP > MBC > URE > silt > SWC > MBP > sand > BD > clay > ALP > AK > SOC > TN > TC > Fungal diversity > Bacterial diversity. MBN, TP, MBC, URE, silt, SWC, MBP, sand, BD, clay, ALP, AK, SOC, TN, and TC all fell within 10% of the maximum Norm value. However, because the TP content of the *Picea crassifolia* pure forest showed extremely significant (*p* < 0.01) correlations with MBN, MBC, URE, silt, SWC, MBP, sand, BD, clay, ALP, AK, SOC, TN, and TC, TP was selected for the MDS. The Norm value ranking in PC2 was as follows: AN > AP > pH > EC > SUC > TK > CAT. AN, AP, pH, EC, and SUC fell within 10% difference range of the highest Norm value. Because soil AN exhibited a highly significant correlation (*p* < 0.01) with AP, pH, EC, and SUC, AN was selected for the MDS. The final MDS indicators for the *Picea crassifolia pure forest* was TP and AN.

In the *Betula* pure forest of the study area, the three principal components (PC1, PC2, and PC3) with eigenvalues >1 explained 98.44% of the overall variance (Table S3). The soil indicators in PC1 were TP, TK, AN, AK, MBC, MBN, MBP, SUC, URE, clay, silt, and sand; PC2 included TC, TN, SOC, AP, ALP, CAT, SWC, BD, EC, and pH; PC3 comprised only bacterial diversity and fungal diversity. Similarly, TP from PC1 and BD from PC2 were selected for the MDS. The Norm values for PC3 were ranked as follows: fungal diversity > bacterial diversity. Both indicators fell within 10% of each other in terms of Norm value, and their correlation was not significant. Therefore, both bacterial diversity and fungal diversity were incorporated into the MDS. The resulting MDS for the *Betula* pure forest was TP, BD, bacterial diversity, and fungal diversity.

In the *Juniperus przewalskii* pure forest of the study area, the two principal components (PC1 and PC2) with eigenvalues >1 explained 95.68% of the overall variance (Table S4). The soil indicators in PC1 were TC, TN, TP, SOC, AN, AK, SUC, URE, BD, clay, silt, sand, and bacterial diversity. PC2 included TK, AP, MBC, MBN, MBP, ALP, CAT, SWC, EC, BD, pH, and fungal diversity. Sand from PC1 and SWC from PC2 were incorporated into the MDS. The resulting MDS for the *Juniperus przewalskii* pure forest was sand and SWC.

In the *Pinus tabuliformis* pure forest of the study area, the three principal components (PC1, PC2, and PC3) with eigenvalues >1 explained 99.32% of the overall variance (Table S5). The soil indicators in PC1 were silt, BD, pH, AK, SUC, TP, SWC, AN, TC, SOC, ALP, sand, and TN. PC2 included AP, MBC, MBN, clay, URE, MBP, TK, CAT, and EC. PC3 comprised bacterial diversity and fungal diversity. Similarly, pH from PC1, EC from PC2, and bacterial diversity from PC3 were incorporated into the MDS. The resulting MDS for the *Pinus tabuliformis* pure forest was pH, EC, and bacterial diversity.

Within the mixed conifer–broadleaf forest in the study area, the three principal components (PC1, PC2, and PC3) with eigenvalues >1 explained 98.4% of the overall variance (Table S6). The soil indicators in PC1 were sand, silt, clay, MBP, AN, CAT, AP, SOC, TC, SWC, TN, and MBN. PC2 included MBC, EC, URE, ALP, AK, TK, SUC, TP, BD, and pH. PC3 comprised bacterial and fungal diversity. Sand derived from PC1, BD from PC2, and bacterial and fungal diversity derived from PC3 were incorporated into the MDS. The resulting MDS for the mixed conifer–broadleaf forest was sand, BD, bacterial diversity, and fungal diversity.

In the mixed broadleaved forest of the study area, the three principal components (PC1, PC2, and PC3) with eigenvalues >1 explained 97.08% of the overall variance (Table S7). The soil indicators in PC1 were MBN, silt, AN, URE, MBP, sand, MBC, clay, SUC, AP, pH, TK, and TP. PC2 included EC, ALP, TC, SOC, CAT, BD, SWC, AK, and TN. PC3 comprised bacterial diversity and fungal diversity. MBN from PC1, TC from PC2, and bacterial and fungal diversity from PC3 were incorporated into the MDS. The resulting MDS for the mixed broadleaved forest was MBN, TC, bacterial diversity, and fungal diversity.

#### 3.4.2. SQ Evaluation Based on the MDS

**Figure 6:**
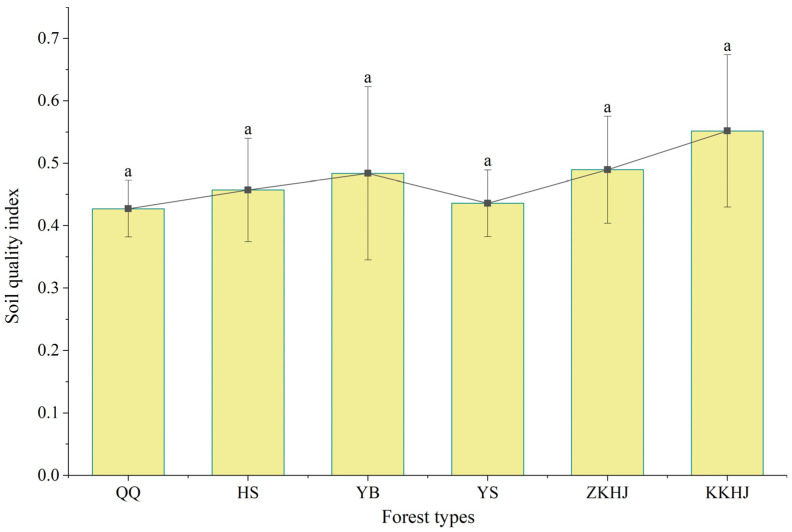
Soil quality index across various forest types. Values sharing the same lowercase letter indicate no significant differences in the soil quality index among different forest types.

For the indicators selected into the MDS for different forest types, PCA was further applied to derive the communalities for the MDS indicator system and to determine the weights of the corresponding indicators (Table 3). The SQI function method was selected to calculate the SQIs for the forest MDS across the study area. As presented in Figure 6, the average SQI for the forested areas along the southern flank of the Qilian Mountains was 0.47, with KKHJ showing the highest SQI (0.55), followed by ZKHJ (0.49), YB (0.48), HS (0.46), YS (0.44), and QQ (0.43). SQ was classified into Grades I–VI, corresponding to index ranges of ≤0.30, (0.30–0.40], (0.40–0.50], (0.50–0.60], (0.60–0.70], and >0.70, respectively. Among these, only the mixed broadleaved forest exhibited an SQ grade of IV (upper-intermediate level), while the remaining five forest types fell into Grade III, thereby indicating an intermediate level.

## 4. Discussion

### 4.1. Analysis of Soil Microbial Composition, Diversity, and Driving Factors Across Various Forest Types

Various vegetation forms selectively shape the abundance and diversity of soil bacterial taxa through differences in litter input, root activity, and associated soil environments [19]. Across the six forest types, fourteen dominant bacterial phyla with relative abundances greater than 1% were identified, with Proteobacteria, Acidobacteriota, Actinobacteriota, and Chloroflexi consistently dominating. In addition, seven dominant fungal phyla were detected, among which Ascomycota and Basidiomycota were the major groups. Although the overall dominance patterns were consistent with previous studies on the southern slope of the Qilian Mountains [13,20], the relative abundances of these microbial groups varied significantly among forest types. These variations were closely linked to differences in soil physicochemical properties (Table 1). Forest types with higher soil organic carbon, total nitrogen, and microbial biomass, such as the mixed forests ZKHJ and KKHJ, supported higher relative abundances of Proteobacteria. This pattern reflects the preference of Proteobacteria for nutrient-rich environments and their important roles in nitrogen cycling, including nitrogen fixation, nitrification, and denitrification [21,22]. In contrast, forest soils with lower nutrient availability and higher bulk density, such as YS, showed increased proportions of Acidobacteriota. This group is well adapted to oligotrophic conditions and is commonly associated with acidic to weakly acidic soils [23]. The relative abundances of Actinobacteriota and Chloroflexi were higher in forest types experiencing lower soil moisture and stronger environmental stress. This trend is consistent with their physiological adaptations to drought, low temperatures, and the decomposition of recalcitrant organic substrates under alpine conditions [24]. Fungal community composition showed similar forest-type-specific responses. Ascomycota were more abundant in forest types with higher soil organic carbon and moisture due to their efficiency in decomposing plant-derived residues [25]. In contrast, Basidiomycota were relatively enriched in forest types with greater moisture availability and organic matter inputs, reflecting their capacity to decompose complex compounds such as lignin and their key role in maintaining soil carbon and nitrogen cycling [26].

Differences in soil physicochemical conditions among forest types strongly regulated microbial alpha diversity in this alpine region. Lower soil water content and reduced nutrient availability in YS, together with higher soil pH and bulk density, likely constrained microbial habitat suitability and resulted in simplified bacterial and fungal communities [27,28]. In contrast, HS and YB exhibited higher bacterial richness and diversity under more favorable moisture and nutrient conditions. Mixed forest stands (ZKHJ and KKHJ) consistently supported higher bacterial and fungal diversity, emphasizing the importance of vegetation complexity in shaping soil microbial assemblages. Diverse root systems and litter inputs in mixed forests improve soil structure, enhance moisture retention, and increase organic matter availability, thereby providing a wider range of nutrient substrates for microbial growth [29]. Bacterial and fungal diversity in KKHJ slightly exceeded that in ZKHJ, suggesting that differences in tree species composition may further stabilize soil environments and promote microbial diversity [30]. Fungal diversity showed patterns similar to those of bacterial communities, with mixed forests exhibiting higher diversity, YS showing the lowest values, and QQ and HS displaying intermediate levels. OTU-based NMDS analysis further supported these patterns, as microbial communities in YB and YS were clearly separated from those in other forest types, while ZKHJ and KKHJ partially overlapped, indicating comparable microhabitat conditions and reinforcing the role of mixed forests in supporting stable and diverse soil microbial communities.

In forest soil ecology research, bacteria and fungi serve as key members of soil microbial communities. Their diversity is comprehensively regulated by various environmental, edaphic, enzymatic, and vegetation biotic factors. SEM results demonstrated that environmental factors predominantly promoted soil bacterial and fungal diversity across forest types in the Qilian Mountains. This result suggested that macro-topographic elements, such as elevation, slope, and aspect, indirectly influenced microbial assemblage patterns through regulating soil temperature, moisture, and organic matter accumulation. This finding aligned with the results of Bahram et al. [31], which identified environmental gradients, specifically temperature and moisture conditions linked to increasing elevation, as dominant drivers influencing the spatial differentiation of microbial diversity in mountain forest ecosystems. The low temperatures and arid stress at high elevations in the Qilian Mountains exerted a strong environmental selection pressure on microbial communities, promoting the differentiation and adaptation of stress-tolerant bacterial (e.g., *Actinobacteriota* and *Chloroflexi*) and fungal (e.g., *Ascomycota*) phyla [32]. Conversely, soil chemical properties negatively impacted bacterial and fungal diversity, indicating that high nutrient levels did not necessarily promote community diversity and may reduce community evenness [33]. Furthermore, environments with low pH and high EC may restrict the survival of certain sensitive microbial communities, leading to functional redundancy in community structure. Soil pH and nutrient status significantly influenced bacterial diversity, while their effects on fungi were comparatively weaker, thereby reflecting the greater ecological tolerance of fungi [34]. The model analysis also indicated that soil enzyme activities (SUC, URE, ALP, and CAT) had weak positive effects on bacterial diversity, suggesting a potential role of enzyme activity in promoting microbial metabolic functions and maintaining diversity. Although the effects were not statistically significant, this trend aligned with the findings of Luo et al. [35], positing that higher enzyme activity represented stronger soil carbon and nitrogen cycling potential, thereby providing bacteria with a more stable substrate supply. Fungal diversity exhibited a negative response to enzyme activity, likely due to certain fungi (e.g., *Basidiomycota*) experiencing reduced competitiveness in high-enzyme-activity environments, where their ecological niches were occupied by more metabolically flexible bacterial groups. Biotic factors (forest type, tree height, DBH, and crown spread) significantly negatively impacted fungal diversity, indicating that different vegetation structures selectively influenced fungal community assembly. Mixed forests often promoted fungal community complexity due to higher root exudate diversity, whereas the chemical composition of litter from single-species coniferous forests (e.g., YS) may inhibit certain fungal growth [36]. In contrast, bacterial communities demonstrated weaker responses to these biotic factors, reflecting their greater adaptability to microenvironmental changes and random dispersal characteristics [37]. Overall, the SEM findings from this study revealed multidimensional mechanisms driving soil microbial diversity along the southern flank of the Qilian Mountains. Specifically, environmental gradients represented the most significant positive factor, whereas soil chemical properties and biotic factors partially suppressed community diversity. Enzyme activity and physical properties exerted relatively indirect effects. These findings were consistent with the general patterns observed in most mountain ecosystems, indicating that microbial diversity in high-altitude arid regions was shaped by the dual influences of environmental filtering and resource limitations [31].

### 4.2. SQ Evaluation Across Different Forest Types

Key soil physicochemical properties and microbial diversity jointly governed soil quality variation across forest types in this alpine region. Indicators related to phosphorus availability, soil structure, texture, and microbial diversity were repeatedly selected in the minimum data sets, highlighting soil TP, bulk density, sand content, and bacterial and fungal diversity as the most influential factors shaping soil quality across the southern flank of the Qilian Mountains. TP, as a vital marker of soil nutrient reserves, significantly impacted microbial community diversity, particularly bacterial and fungal diversity. Previous research has indicated that soil TP serves as a major determinant of microbial activity and community structure at broader regional scales [38]. In the context of nutritional ecology, phosphorus-limited conditions may result in competition for limited resources, potentially leading to simplified microbial community structures. Conversely, moderate soil phosphorus levels support greater ecological niche diversity and facilitate the coexistence of multiple microbial species [39]. This result showed that soil TP levels in both the QQ and HS pure forests were moderate, which may explain why TP emerged as a key indicator influencing SQ in these ecosystems. Soil BD reflects soil compaction, pore structure, and air/water permeability. These physical conditions directly influence microbial habitat space and dispersal capacity. For instance, Peng et al. [40] found that soil BD negatively impacted plant root and soil animal activity, thereby indirectly affecting microbial diversity. In this study, soil BD, bacterial diversity, and fungal diversity simultaneously served as key indicators of SQ in both the HS pure forest and ZKHJ mixed forest, highlighting a significant association between soil BD and soil microbial diversity. Therefore, effectively managing forest soil BD and enhancing microbial diversity leads to increased availability of ecological niches and improved capacity for maintaining SQ stability. In addition, soil sand content influences water retention, nutrient conservation, pore structure, and microhabitat distribution, thereby influencing SQ. Chau et al. [41] observed that coarse-textured soils, characterized by high sand content, exhibited increased bacterial species richness. This was attributed to the coarse structure, which created “isolated water film” microenvironments that promote microbial habitat heterogeneity. In this study, the KKHJ mixed forest demonstrated the highest soil sand content and relative SQI, highlighting the pivotal regulatory role of soil texture on microbial assemblages and SQ.

The average SQI for forests on the southern slope of the Qilian Mountains was 0.47, indicating an overall intermediate SQ level. This finding aligned with that of Jiang et al. [42] regarding the SQ (0.48) of the forests on the northern slope of the Qilian Mountains. The SQIs for different forest types were ranked as follows: KKHJ (0.55) > ZKHJ (0.49) > YB (0.48) > HS (0.46) > YS (0.44) > QQ (0.43). This indicated that mixed forests generally exhibited higher SQ than pure forests. In contrast, the SQIs of the YS and QQ pure forests were lower, likely due to reduced soil nutrient accumulation in these areas. Mixed forests often enhanced SQ more effectively owing to root and litter diversity [43]. The KKHJ and ZKHJ forests shared common MDS indicators, such as sand, BD, bacterial diversity, fungal diversity, MBN, and TC. Based on the soil physicochemical properties presented in Table 2, KKHJ and ZKHJ exhibited the highest TC (161.62 and 161.97 g/kg, respectively), MBN (115.27 and 138.36 g/kg, respectively), TN, and SOC contents, suggesting that mixed forests possessed enhanced capacities for organic matter accumulation and nutrient retention. This was possibly because diverse quantities and chemical compositions of litter from various tree species could facilitate decomposition and soil carbon, nitrogen, and phosphorus cycling [44]. Moreover, the higher SWC and sand content, along with lower BD levels in mixed forests, provided favorable aeration and moisture conditions for microbial activity, thereby promoting soil microbial diversity and SQ [45]. Conversely, the YS and QQ pure forests displayed lower SQIs, with nutrient contents such as TC, TN, and SOC being at the lowest levels (e.g., the YS pure forest demonstrated a TC level of only 55.49 g/kg and an SOC level of merely 40.46 g/kg), while BD remained elevated (1.52 and 1.18 g/cm^3^, respectively). This suggested soil compaction, poor aeration, and limited microbial activity. Notably, YS exhibited an SWC level as low as 20.36% and a pH as high as 7.97. This, combined with drought, nutrient deficiency, and acid-base imbalance, collectively suppressed soil microbial diversity and nutrient cycling efficiency [38]. In contrast, YB and HS demonstrated intermediate levels. Although their SOC and TN contents were relatively high, their SWC and TP levels were slightly lower, indicating favorable carbon and nitrogen accumulation but persistent phosphorus limitation. In summary, mixed forests on the southern slope of the Qilian Mountains exhibited superior soil physicochemical structure and conditions conducive to diverse microbial communities, resulting in enhanced SQ and ecological functional stability compared with single-species forests. This trend validated the positive feedback mechanism linking vegetation diversity, SQ, and microbial activity [33,46].

## 5. Conclusions

This study examined bacterial and fungal diversity within soils across various forest types along the southern flank of the Qilian Mountains and analyzed the corresponding influencing factors. Based on MDS, the SQ of four pure (QQ, HS, YB, and YS) and two mixed (ZKHJ and KKHJ) forests was comprehensively evaluated. Our findings indicated that the prevailing bacterial lineages across the forests on the southern slope of the Qilian Mountains were dominated by *Proteobacteria*, *Acidobacteriota*, *Actinobacteriota*, and *Chloroflexi*, while the fungal communities were primarily structured by *Ascomycota* and *Basidiomycota*. The mixed stands (ZKHJ and KKHJ) showed higher bacterial and fungal diversity due to their diverse plant roots and litter, improved soil structures, and more favorable moisture conditions. In contrast, YS showed lower bacterial and fungal species richness and diversity. Therefore, mixed forests offer a more stable and diverse habitat for microbial communities. SEM revealed environmental gradients as the most significant positive factor, while soil chemical properties and biotic factors partially suppressed community diversity. Furthermore, enzyme activity and physical properties exerted more indirect effects. The average SQI for forests on the southern slope of the Qilian Mountains was 0.47, indicating an overall intermediate SQ. Among different forest types, mixed forests generally exhibited higher SQ than pure forests. Compared with single-species pure forests, mixed forests on the southern slope of the Qilian Mountains possessed better soil physicochemical structures and conditions for diverse microbial community formation, resulting in higher SQ and ecological functional stability.

## Figures and Tables

**Table 1:** Key site characteristics of forest stands across elevation gradients.

Plot ID	Forest Type	Elevation (m)	Mean Vegetation Cover (%)	Mean Diameter at Breast Height (cm)	Mean Tree Height (m)	Average East–West Crown Width (m)	Average North–South Crown Width (m)	Aspect (°)	Slope (°)	Vegetation Composition
I	Pure *Picea crassifolia* forest	2328	45	18.93	15.66	2.43	2.93	23	16	The canopy is formed primarily by *Picea crassifolia*, with shrubs including *Ribes spinosum*, *Lonicera tangutica*, and *Rosa acicularis*. The herb layer features *Fragaria orientalis*, *Dryopteris* spp., and *Vaccinium vitis-idaea*.
II	Pure *Betula* forest	2941	72	8.21	7.47	3.05	3.35	333	23.22	The overstory is dominated by *Betula platyphylla*, with shrubs including Lonicera tangutica, Rhododendron przewalskii, and Rosa sericea. Herbaceous cover consists of *Fragaria orientalis*, *Dryopteris* spp., and *Rubus* spp.
III	Pure *Juniperus przewalskii* forest	3255	60	14.81	5.79	2.24	2.77	244	14.85	The upper layer is dominated by *Juniperus przewalskii*, with shrubs such as *Potentilla fruticosa*, *Berberis* spp., and *Sempervivella* spp. Herbaceous species present include *Fragaria orientalis*, *Polygonum viviparum*, and *Poa pratensis*.
IV	Pure *Pinus tabuliformis* forest	2378	75	15.36	13.63	2.16	2.51	64	20.5	The canopy is dominated by *Pinus tabuliformis*, with *Dasiphora nivea* in the shrub layer and *Fragaria orientalis* and *Carex* spp. in the herb layer.
V	Mixed coniferous-broadleaved forest	2512	70	22.91	19.26	2.47	3.32	341	21.77	The canopy is dominated by *Betula albo-sinensis* and *Picea crassifolia*. Shrubs are mainly *Lonicera tangutica* and *Rosa sericea*, while the herb layer is characterized by *Dryopteris* spp.
VI	Mixed broadleaved forest	2559	75	25.08	15.62	2.66	3.61	56	24.31	The canopy is formed mainly by *Betula platyphylla* and *Betula albo-sinensis*. The shrub layer consists chiefly of *Lonicera tangutica*, and the herb layer is dominated by *Vicia amoena*, *Saussurea* spp., and *Thalictrum* spp.

**Table 2:** Comparative physicochemical profiles of soils across the forest types.

Indicators	Pure *Picea crassifolia* Forest	Pure *Betula* Forest	Pure *Juniperus przewalskii* Forest	Pure *Pinus tabuliformis* Forest	Mixed Coniferous-Broadleaved Forest	Mixed Broadleaved Forest
TC (g·kg^–1^)	82.91 ± 12.32 ^bc^	105.22 ± 11.16 ^b^	64.4 ± 3 ^bc^	55.49 ± 12.48 ^c^	161.97 ± 16.55 ^a^	161.62 ± 15.76 ^a^
TN (g·kg^–1^)	5.58 ± 0.28 ^bc^	7.97 ± 0.98 ^b^	5.21 ± 0.26 ^bc^	3.23 ± 0.71 ^c^	12.15 ± 1.28 ^a^	12.76 ± 1.08 ^a^
TP (g·kg^–1^)	0.71 ± 0.05 ^b^	0.72 ± 0.01 ^b^	0.84 ± 0.02 ^a^	0.46 ± 0.02 ^c^	0.77 ± 0.01 ^ab^	0.73 ± 0.03 ^b^
TK (g·kg^–1^)	21.04 ± 0.54 ^b^	18.51 ± 0.08 ^d^	23.89 ± 0.26 ^a^	23.24 ± 0.29 ^a^	19.7 ± 0.33 ^c^	17.74 ± 0.24 ^d^
SOC (g·kg^–1^)	69.33 ± 4.74 ^bc^	101.08 ± 12.01 ^b^	61.81 ± 2.44 ^bc^	40.46 ± 9.33 ^c^	157.99 ± 16.17 ^a^	156.83 ± 14.43 ^a^
AN (mg·kg^–1^)	367.76 ± 21.42 ^b^	470.78 ± 40.85 ^b^	346.58 ± 10.86 ^b^	187.25 ± 12.16 ^c^	673.19 ± 51.97 ^a^	621.44 ± 61.17 ^a^
AP (mg·kg^–1^)	10.82 ± 0.71 ^bc^	9.28 ± 1.19 ^cd^	5.42 ± 0.43 ^d^	5.77 ± 0.39 ^cd^	15.63 ± 2.17 ^ab^	16.74 ± 2.48 ^a^
AK (mg·kg^–1^)	192.57 ± 10.19 ^b^	179.1 ± 13.41 ^b^	220.57 ± 13.2 ^b^	215.27 ± 11.81 ^b^	157.4 ± 4.93 ^b^	280.07 ± 18.72 ^a^
MBC (mg·kg^–1^)	880.94 ± 74.06 ^b^	846.16 ± 99.82 ^b^	534.1 ± 31.88 ^b^	730.82 ± 82.14 ^b^	1822.92 ± 177.32 ^a^	1602.73 ± 176.97 ^a^
MBN (mg·kg^–1^)	58.93 ± 5.17 ^b^	54.19 ± 6.76 ^b^	36.48 ± 6.37 ^b^	53.56 ± 6.79 ^b^	138.36 ± 19.61 ^a^	115.27 ± 18.74 ^a^
MBP (mg·kg^–1^)	23.39 ± 2.09 ^bc^	22.96 ± 2.03 ^bc^	12.62 ± 0.79 ^c^	25.27 ± 2.73 ^b^	60.09 ± 4.19 ^a^	49.57 ± 3.28 ^a^
SUC (mg·d^–1^·g^–1^)	98.1 ± 10.88 ^bc^	117.22 ± 11.51 ^b^	126.44 ± 17.8 ^b^	66.05 ± 8.48 ^c^	105.24 ± 5.65 ^b^	173.06 ± 6.1 ^a^
URE (mg·d^–1^·g^–1^)	1.68 ± 0.23 ^b^	0.85 ± 0.06 ^c^	0.88 ± 0.09 ^c^	2.34 ± 0.27 ^a^	1.41 ± 0.21 ^bc^	1.87 ± 0.25 ^ab^
ALP (mg·d^–1^·g^–1^)	9.06 ± 0.5 ^bc^	10.11 ± 0.37 ^b^	6.54 ± 0.3 ^d^	7.66 ± 0.58 ^cd^	10.5 ± 0.71 ^b^	12.51 ± 0.89 ^a^
CAT (mg·d^–1^·g^–1^)	7.48 ± 0.16 ^b^	5.74 ± 0.22 ^c^	5.78 ± 0.02 ^c^	8.49 ± 0.26 ^a^	6.12 ± 0.39 ^c^	6.42 ± 0.25 ^c^
SWC (%)	32.32 ± 1.68 ^c^	46.44 ± 2.39 ^b^	44.24 ± 3.13 ^c^	20.36 ± 2.01 ^d^	54.69 ± 3.03 ^b^	56.06 ± 2.09 ^a^
BD (g·cm^–3^)	1.18 ± 0.19 ^ab^	1.05 ± 0.12 ^bc^	1.35 ± 0.03 ^ab^	1.52 ± 0.06 a	0.7 ± 0.03 ^d^	0.84 ± 0.08 ^cd^
EC (μs·cm^–1^)	171.3 ± 6.25 ^b^	221.33 ± 16.25 ^a^	91.13 ± 6.96 ^c^	165.57 ± 6.26 ^b^	240 ± 10.15 ^a^	254 ± 13.58 ^a^
pH	7.41 ± 0.1 ^b^	6.42 ± 0.08 ^de^	6.05 ± 0.21 ^e^	7.89 ± 0.19 a	6.67 ± 0.15 ^cd^	6.99 ± 0.08 ^bc^
Clay (%)	29.92 ± 1.25 ^a^	23.15 ± 1.02 ^ab^	27.84 ± 2.38 ^a^	26.57 ± 1.51 ^ab^	27.36 ± 3.67 ^a^	19.11 ± 1.93 ^b^
Silt (%)	39.24 ± 0.74 ^abc^	36.67 ± 1.52 ^bc^	40.12 ± 0.44 ^ab^	42.92 ± 0.47 ^a^	36.7 ± 1.05 ^bc^	34.16 ± 1.07 ^c^
Sand (%)	30.84 ± 1.98 ^b^	40.18 ± 1.53 ^ab^	32.04 ± 1.77 ^b^	30.51 ± 1.83 ^b^	35.95 ± 1.09 ^ab^	46.73 ± 1.57 ^a^

Note: TC—total carbon; TN—total nitrogen; TP—total phosphorus; TK—total potassium; SOC—soil organic carbon; AN—alkali-hydrolyzable nitrogen; AP—available phosphorus; AK—available potassium; MBC—microbial biomass C; MBN—microbial biomass N; MBP—microbial biomass P; SUC—ucrase; URE—urease; ALP—alkaline phosphatase; CAT—catalase; SWC—soil water content; BD—bulk density; EC—electrical conductivity; pH—soil acidity/alkalinity; Clay, Silt, and Sand—fractions representing soil particle-size composition. Sample size: *n* = 9. Values are expressed as mean ± standard error. Different lowercase letters denote statistically significant differences among forest types at *p* < 0.05. The same notation is used in subsequent tables.

**Table 3:** Communality and weight of the MDS.

Evaluation Index	Pure *Picea * *crassifolia* Forest		Pure *Betula* Forest		Pure *Juniperus * *przewalskii* Forest		Pure *Pinus * *tabuliformis* Forest		Mixed Coniferous- Broadleaved Forest		Mixed Broadleaved Forest	
	Communality	Weight	Communality	Weight	Communality	Weight	Communality	Weight	Communality	Weight	Communality	Weight
TC											0.53	0.25
TP	0.88	0.50	0.95	0.32								
AN	0.88	0.50										
MBN											0.63	0.29
SWC					0.52	0.5						
BD			0.53	0.18					0.65	0.22		
EC							0.48	0.29				
pH							0.71	0.43				
Sand					0.52	0.5			0.72	0.25		
Bacterial diversity			0.69	0.23			0.47	0.28	0.78	0.27	0.71	0.33
Fungal diversity			0.79	0.27					0.75	0.26	0.82	0.38

## Data Availability

All relevant data are provided within the paper and its Supporting Information files.

## Supplementary Materials

The following supporting information can be downloaded at https://www.mdpi.com/article/10.3390/plants15020315/s1: Table S1: Summary of all significant direct paths in the structural equation models (SEM); Table S2: PCA results of SQ indicators in Picea crassifolia pure forest; Table S3: PCA results of SQ indicators in the Betula pure forest; Table S4: PCA results of SQ indicators in Juniperus przewalskii pure forest; Table S5: PCA results of SQ indicators in Pinus tabuliformis pure forest; Table S6: PCA results of SQ indicators in mixed coniferous-broadleaved forest; Table S7: PCA results of SQ indicators in mixed broadleaved forest.
