# Supplementary material for: Influence of Alpine Forest Types on Soil Microbial Diversity and Soil Quality
Source: Plants (Basel). 2026 Jan 21;15(2):315. doi: 10.3390/plants15020315 (PMC12845449; doi:10.3390/plants15020315)
Supplement: Supplementary file 1 [file plants-15-00315-s001.zip › plants-4065153-supplementary.pdf]

plants

MDPI

Supplementary Materials:

Table S1. Summary of all significant direct paths in the structural equation models (SEM).

SEM type	Response variable	Predictor variable	Path coefficient (λ)	Significance level	Effect direction
Bacterial SEM	Soil bacterial diversity	Environmental factors	0.8	***	Positive
	Soil bacterial diversity	Chemical factors	−0.74	**	Negative
	Soil enzyme activity	Physical factors	−0.69	***	Negative
	Chemical factors	Environmental factors	0.22	***	Positive
	Biological factors	Environmental factors	0.63	***	Positive
	Physical factors	Biological factors	−0.53	**	Negative
Fungal SEM	Soil fungal diversity	Environmental factors	0.64	***	Positive
	Soil fungal diversity	Biological factors	−0.37	**	Negative
	Soil enzyme activity	Physical factors	−0.80	***	Negative
	Chemical factors	Environmental factors	0.21	***	Positive
	Biological factors	Environmental factors	0.58	***	Positive
	Physical factors	Biological factors	−0.49	**	Negative

Table S2. PCA results of SQ indicators in *Picea crassifolia* pure forest.

Indicator	Principal component (PC)		Grouping	Norm value
	PC1	PC2		
TC (g/kg)	0.76	0.65	1	3.47
TN (g/kg)	0.80	0.60	1	3.52
TP (g/kg)	0.99	0.15	1	3.81
TK (g/kg)	0.11	−0.98	2	2.84
SOC (g/kg)	0.81	0.58	1	3.54
AN (mg/kg)	0.67	0.74	2	3.34
AP (mg/kg)	0.65	0.76	2	3.31
AK (mg/kg)	−0.85	−0.52	1	3.61
MBC (mg/kg)	0.98	0.19	1	3.80
MBN (mg/kg)	0.99	−0.01	1	3.82
MBP (mg/kg)	0.92	0.38	1	3.72
SUC (mg/d/g)	0.46	0.89	2	3.09
URE (mg/d/g)	0.98	−0.14	1	3.80
ALP (mg/d/g)	0.86	0.50	1	3.62
CAT (mg/d/g)	0.10	−0.98	2	2.84
SWC (%)	0.94	0.34	1	3.74
BD (g/cm³)	−0.91	−0.41	1	3.70
EC (μS/cm)	0.49	0.87	2	3.11
pH	−0.58	−0.81	2	3.23
Clay (%)	−0.89	−0.45	1	3.67
Silt (%)	−0.94	−0.33	1	3.75
Sand (%)	0.92	0.40	1	3.70

Bacterial diversity	−0.56	0.55	1	2.66
Fungal diversity	−0.70	0.04	1	2.71
Eigenvalue	14.81	8.18		
Variance contribution rate (%)	77.81	17.96		
Cumulative contribution rate (%)	77.81	95.77		

Table S3. PCA results of SQ indicators in the *Betula* pure forest

Indicator	Principal component (PC)			Grouping	Norm value
	PC1	PC2	PC3		
TC (g/kg)	0.58	0.80	0.14	2	3.22
TN (g/kg)	0.66	0.74	0.14	2	3.26
TP (g/kg)	−1.00	0.06	0.00	1	3.48
TK (g/kg)	0.77	0.63	0.12	1	3.32
SOC (g/kg)	0.57	0.81	0.15	2	3.21
AN (mg/kg)	0.81	0.57	0.11	1	3.35
AP (mg/kg)	0.55	0.82	0.15	2	3.20
AK (mg/kg)	−0.76	0.64	0.10	1	3.32
MBC (mg/kg)	0.92	0.38	0.08	1	3.42
MBN (mg/kg)	0.99	0.12	0.03	1	3.47
MBP (mg/kg)	0.93	0.35	0.07	1	3.43
SUC (mg/d/g)	0.96	0.28	0.06	1	3.45
URE (mg/d/g)	−0.82	0.57	0.09	1	3.35
ALP (mg/d/g)	−0.08	0.98	0.17	2	3.08
CAT (mg/d/g)	0.43	0.89	0.16	2	3.16
SWC (%)	0.40	0.90	0.16	2	3.15
BD (g/cm^3)	−0.21	−0.96	−0.17	2	3.09
EC (μS/cm)	−0.34	0.93	0.16	2	3.13
pH	−0.59	−0.79	−0.14	2	3.22
Clay (%)	−0.93	−0.37	−0.08	1	3.43
Silt (%)	−0.97	−0.22	−0.05	1	3.46
Sand (%)	0.96	0.29	0.06	1	3.45
Bacterial diversity	0.12	0.16	0.89	3	1.35
Fungal diversity	0.02	−0.30	−0.84	3	1.47
Eigenvalue	12.12	9.70	1.80		
Variance contribution rate (%)	67.15	25.95	5.34		
Cumulative contribution rate (%)	67.15	93.10	98.44		

Table S4. PCA results of SQ indicators in *Juniperus przewalskii* pure forest

Indicator	Principal component (PC)		Grouping	Norm value
	PC1	PC2		
TC (g/kg)	1.00	0.08	1	3.53
TN (g/kg)	0.98	−0.21	1	3.51
TP (g/kg)	0.86	−0.50	1	3.45
TK (g/kg)	−0.55	−0.84	2	3.33
SOC (g/kg)	1.00	0.07	1	3.52
AN (mg/kg)	0.90	−0.44	1	3.47
AP (mg/kg)	0.34	−0.94	2	3.27
AK (mg/kg)	0.87	−0.49	1	3.45
MBC (mg/kg)	0.39	0.92	2	3.28
MBN (mg/kg)	−0.12	0.99	2	3.24
MBP (mg/kg)	−0.22	0.97	2	3.25
SUC (mg/d/g)	0.78	0.63	1	3.42
URE (mg/d/g)	0.81	−0.58	1	3.43
ALP (mg/d/g)	−0.35	0.93	2	3.27
CAT (mg/d/g)	0.65	−0.76	2	3.36
SWC (%)	0.03	−1.00	2	3.24
BD (g/cm^3)	0.95	0.31	1	3.50

EC (μS/cm)	−0.05	1.00	2	3.24
pH	0.68	0.74	2	3.37
Clay (%)	−0.99	−0.16	1	3.52
Silt (%)	−0.93	0.37	1	3.49
Sand (%)	1.00	0.08	1	3.53
Bacterial diversity	0.76	−0.28	1	2.81
Fungal diversity	0.14	−0.59	2	1.97
Eigenvalue	12.45	10.51		
Variance contribution rate (%)	55.78	39.90		
Cumulative contribution rate (%)	55.78	95.68		

Table S5. PCA results of SQ indicators in *Pinus tabuliformis* pure forest

Indicator	Principal component (PC)			Grouping	Norm value
	PC1	PC2	PC3		
TC (g/kg)	0.73	0.67	−0.11	1	3.30
TN (g/kg)	0.72	0.68	−0.12	1	3.30
TP (g/kg)	0.82	0.56	−0.11	1	3.34
TK (g/kg)	−0.48	−0.87	0.12	2	3.23
SOC (g/kg)	0.73	0.67	−0.11	1	3.30
AN (mg/kg)	0.77	0.63	−0.11	1	3.32
AP (mg/kg)	0.69	0.72	−0.12	2	3.29
AK (mg/kg)	0.96	0.27	−0.09	1	3.39
MBC (mg/kg)	0.62	0.78	−0.12	2	3.27
MBN (mg/kg)	0.61	0.79	−0.12	2	3.26
MBP (mg/kg)	0.55	0.83	−0.12	2	3.25
SUC (mg/d/g)	0.94	0.32	−0.09	1	3.39
URE (mg/d/g)	−0.50	0.86	−0.05	2	3.25
ALP (mg/d/g)	0.73	0.68	−0.11	1	3.30
CAT (mg/d/g)	−0.18	−0.98	0.11	2	3.19
SWC (%)	0.78	0.62	−0.11	1	3.32
BD (g/cm^3)	−0.99	−0.10	0.08	1	3.41
EC (μS/cm)	0.14	0.98	−0.11	2	3.18
pH	0.96	0.27	−0.09	1	3.40
Clay (%)	−0.58	−0.81	0.12	2	3.26
Silt (%)	−1.00	-0.03	0.07	1	3.42
Sand (%)	0.73	0.68	−0.11	1	3.30
Bacterial diversity	−0.33	−0.02	0.90	3	1.68
Fungal diversity	0.07	−0.25	0.92	3	1.53
Eigenvalue	11.72	10.21	1.91		
Variance contribution rate (%)	78.51	14.37	6.45		
Cumulative contribution rate (%)	78.51	92.88	99.32		

Table S6. PCA results of SQ indicators in mixed coniferous-broadleaved forest

Indicator	Principal component (PC)			Grouping	Norm value
	PC1	PC2	PC3		
TC (g/kg)	0.87	0.50	−0.06	1	3.41
TN (g/kg)	0.85	0.53	−0.07	1	3.40
TP (g/kg)	0.36	0.93	−0.06	2	3.22
TK (g/kg)	−0.38	−0.92	0.06	2	3.23
SOC (g/kg)	0.89	0.45	−0.06	1	3.43
AN (mg/kg)	0.94	−0.35	−0.03	1	3.45
AP (mg/kg)	0.92	0.38	−0.06	1	3.45
AK (mg/kg)	−0.38	0.92	−0.02	2	3.23
MBC (mg/kg)	0.70	0.71	−0.07	2	3.34
MBN (mg/kg)	0.84	0.54	−0.07	1	3.40
MBP (mg/kg)	0.98	−0.21	−0.04	1	3.48
SUC (mg/d/g)	0.37	−0.93	0.02	2	3.23

URE (mg/d/g)	0.43	−0.90	0.02	2	3.24
ALP (mg/d/g)	0.40	0.91	−0.06	2	3.23
CAT (mg/d/g)	−0.93	0.37	0.03	1	3.45
SWC (%)	0.85	0.53	−0.07	1	3.41
BD (g/cm^3)	−0.16	−0.99	0.05	2	3.19
EC (μS/cm)	0.61	0.79	−0.07	2	3.30
pH	−0.04	1.00	−0.04	2	3.18
Clay (%)	−0.99	0.14	0.04	1	3.48
Silt (%)	−1.00	−0.04	0.05	1	3.49
Sand (%)	1.00	−0.07	−0.05	1	3.49
Bacterial diversity	−0.46	0.22	0.74	3	1.92
Fungal diversity	0.14	−0.39	0.81	3	1.62
Eigenvalue	12.21	10.14	1.26		
Variance contribution rate (%)	57.92	35.67	4.81		
Cumulative contribution rate (%)	57.92	93.59	98.40		

Table S7. PCA results of SQ indicators in mixed broadleaved forest

Indicator	Principal component (PC)			Grouping	Norm value
	PC1	PC2	PC3		
TC (g/kg)	−0.22	0.97	0.04	2	3.17
TN (g/kg)	0.02	1.00	0.05	2	3.15
TP (g/kg)	−0.84	0.54	0.02	1	3.40
TK (g/kg)	−0.85	−0.52	−0.03	1	3.41
SOC (g/kg)	−0.19	0.98	0.04	2	3.16
AN (mg/kg)	0.99	0.14	0.01	1	3.49
AP (mg/kg)	0.91	0.42	0.02	1	3.44
AK (mg/kg)	−0.09	0.99	0.05	2	3.15
MBC (mg/kg)	0.96	0.28	0.02	1	3.47
MBN (mg/kg)	1.00	0.03	0.01	1	3.50
MBP (mg/kg)	0.98	−0.18	0.00	1	3.49
SUC (mg/d/g)	−0.94	0.33	0.01	1	3.46
URE (mg/d/g)	0.99	0.15	0.01	1	3.49
ALP (mg/d/g)	−0.26	0.96	0.04	2	3.18
CAT (mg/d/g)	−0.15	−0.99	−0.05	2	3.16
SWC (%)	−0.08	1.00	0.05	2	3.15
BD (g/cm^3)	−0.11	−0.99	−0.05	2	3.15
EC (μS/cm)	0.52	0.85	0.04	2	3.25
pH	−0.89	0.46	0.02	1	3.43
Clay (%)	−0.94	0.34	0.01	1	3.46
Silt (%)	−1.00	0.06	0.00	1	3.50
Sand (%)	0.98	−0.21	−0.01	1	3.48
Bacterial diversity	−0.29	0.26	0.65	3	1.47
Fungal diversity	−0.26	0.04	−0.82	3	1.26
Eigenvalue	12.24	9.95	1.11		
Variance contribution rate (%)	51.80	40.81	4.47		
Cumulative contribution rate (%)	51.80	92.61	97.08		

https://doi.org/10.3390/plants15020315
